# Inoculation with Arbuscular Mycorrhizal Fungi Supports the Uptake of Macronutrients and Promotes the Growth of *Festuca ovina* L. and *Trifolium medium* L., a Candidate Species for Green Urban Infrastructure

**DOI:** 10.3390/plants13182620

**Published:** 2024-09-19

**Authors:** Alicja Szada-Borzyszkowska, Jacek Krzyżak, Szymon Rusinowski, Franco Magurno, Marta Pogrzeba

**Affiliations:** 1 Institute for Ecology of Industrial Areas, 6 Kossutha St., 40-844 Katowice, Poland; a.szada-borzyszkowska@ietu.pl (A.S.-B.); j.krzyzak@ietu.pl (J.K.); s.rusinowski@ietu.pl (S.R.); 2 CommLED Solution Sp. z.o.o., 149 Tarnogórska St., 44-100 Gliwice, Poland; 3 Institute of Biology, Biotechnology and Environmental Protection, Faculty of Natural Sciences, University of Silesia in Katowice, 28 Jagiellońska St., 40-032 Katowice, Poland; franco.magurno@us.edu.pl

**Keywords:** indigenous fungi, arbuscular mycorrhiza fungi, nature-based solutions, calcareous grassland species, plant-growth promotion

## Abstract

Green roofs and walls play an important role in promoting biodiversity, reducing the urban heat island effect and providing ecosystem services in urban areas. However, the conditions on green walls/roofs (low nutrient and organic matter content, drought, high temperatures) are often unfavorable for plant growth. Arbuscular mycorrhizal fungi (AMF) can improve the growth and development of plants under stress conditions as they can increase nutrient and water uptake. In a 6-month pot experiment, we investigated the effect of AMF inoculation on the growth and NPK uptake of *Festuca ovina* L. and *Trifolium medium* L., which are used for green roofs and walls. Two variants of mycorrhizal inoculation were used in the experiment: a commercial mycorrhizal inoculant AM Symbivit (Symbiom Ltd., Lanskroun, Czech Republic) and a mycorrhizal inoculant collected from calcareous grassland in the Silesia region (Poland). *Funneliformis mosseae* was the most abundant species in the roots of *F. ovina* and *T. medium* with IM inoculum. In the CM variant, a dominance of *F. mosseae* was observed in the roots of *F. ovina*. In contrast, *Archaeosporaceae* sp. node 317 dominated in the roots of *T. medium*. Both inoculations had a positive effect on the increase in dry weight of the shoots of *T. medium*, but only the commercial inoculum had a positive effect on the growth of *F. ovina*. Both inoculations improved the P uptake by the roots and the P and K uptake into the shoots of *T. medium*. In addition, both inoculations improved the K uptake by the roots of *F. ovina* and the N, P and K uptake into the shoots. In conclusion, both AMF communities included in the inoculations had a positive effect on plant growth and nutrient uptake, but the effect depends on the plant and the mycorrhizal fungus species.

## 1. Introduction

Increasing urbanization and the associated consequences in the form of a reduction in green spaces, the creation of urban heat islands (UHI), the limitation of surface runoff and the decline in biodiversity are driving global environmental change [1]. Nature-based solutions such as green roofs or green walls are important ecological spaces in urban environments [2] and offer the potential to improve biodiversity, reduce the impact of urban heat islands, improve the esthetic value of cities, provide ecosystem services in urban areas and influence public health [3,4,5,6]. The environment of green roofs and walls is exposed to many stress factors, e.g., the substrates contain less organic matter and nutrients compared to natural soils, excessive drought and high temperatures [7,8]. Therefore, it is important to select plants for green roofs/walls that have a high tolerance to abiotic stress factors [9,10,11], which severely affect plant growth and productivity [12,13]. To promote biodiversity, green roofs or walls are planted with native plant species [14]. According to Krzyżak et al. [15], the suitable plant species for cultivation on green roofs/walls under Polish climate conditions are plants from natural habitats, especially calcareous grasslands, for example, *Festuca ovina*, *Trifolium medium*, *Potentilla reptans* L. and *Carex flacca* Schreb.

In the presence of biotic and abiotic stress, one of the plant strategies to cope with stress factors is the symbiosis of plants with soil microorganisms [16,17,18].

Arbuscular mycorrhizal fungi (AMF), belonging to the phylum *Mucoromycota* and subphylum *Glomeromycotina*, develop mutualistic symbiotic associations with about 80–90% of land plant species [19,20,21]. The relationship between AMFs and plants leads to promoting plant growth and development [22,23,24], improving plant adaptation to environmental stressors [25] and a bilateral exchange of soil resources such as phosphorus, nitrogen and water in exchange for photosynthetic products [26,27,28]. Most plant species growing on green roofs form associations with AM fungi [29,30]. Studies report that green roof substrates are not specifically inoculated with AMF on a commercial scale [2,8] and the use of AMF inoculation in green roof design has not been well studied [5]. In the context of plant growth on green roofs/walls, the symbiosis of plants with AMF can bring a number of benefits, in particular, better establishment of vegetation, higher tolerance of plants to drought and low nutrient levels [31,32,33]. The studies on the role of AMF on green roofs were carried out with single AMF isolates [34,35]. However, this does not reflect the natural conditions under which AMFs occur as a multispecies community in which they compete for space, carbon resources, nutrients and water [36,37,38]. In this study, we investigated the effects of two variants of inoculation with two different AMF communities on the growth and development of *F. ovina* and *T. medium* as potential candidates for cultivation on green roofs or walls. Considering that the benefit to plants in terms of nutrient acquisition is highly dependent on mycorrhizal associations [21], host plant species/genotype and fungal species [33,39,40,41], three hypotheses were proposed: AMF inoculation produced from calcareous grassland (IM) will improve plant growth (1) and NPK uptake (2) more effectively than commercial inoculation (CM); (3) IM inoculation would characterize plant AMF community by higher richness. However, it should be noted that different characteristics of the green roof/wall may have an influence on the frequency of AMF, e.g., the age of the green roof, the N and P concentration in the substrate of the green roof/wall, the height of the green roof or the height of the building on which the green roof is located [2]. Therefore, the research is preliminary and requires a more detailed and long-term analysis of the effectiveness of microbial inoculants in the specific environment for which they are intended.

## 2. Results

### 2.1. Plant Growth and Nutrient Aquisition

The dry weight of the shoots and the NPK content in the shoots and roots of *F. ovina* and *T. medium* are shown in Table 1.

Both mycorrhizal inoculants resulted in an approximately 7% higher P content in the shoot of *F. ovina* compared to the corresponding control (C). Moreover, commercial inoculum (CM) increased the K and N content by about 42–60% compared to C. Slightly different trends were observed in the roots, where the P and N content did not differ significantly between the treatments and the control. The K content was 2-fold and 50% higher in inoculum from calcareous grassland (IM) and CM, respectively. Shoot dry weight did not increase significantly under IM inoculation, while CM inoculation of *F. ovina* resulted in an almost 2-fold increase in shoot dry weight compared to the other treatments.

CM inoculation of *T. medium* led to a significant increase in P and K content in the shoots, and this increase was more pronounced: the P and K content was almost 5 and more than 2 times higher, respectively, compared to the control. This inoculant in *T. medium* did not lead to an increase in shoot N content, which was also true for IM. In contrast to the results of *F. ovina*, IM increased the P and K content in the shoots to the level of the CM treatment. No significant differences were found in root nutrient content between the control and inoculation. The only difference was found in P, where both inoculants increased the P content in the roots by almost 2-fold. Interestingly, the dry weight of the shoots of *T. medium* was highest in the IM treatment, and the reported values were 2 and 3 times higher in the CM and IM treatments, respectively, than in C. The P and K content in the shoots of *T. medium* was statistically significantly different between C and the other variants. In the mycorrhizal variants, the P content was about 4.5 times higher than in the C variant. In contrast, the K content in the mycorrhiza treatments was 2 times higher than in the C variant. The P content in the roots of *T. medium* was statistically significantly different between the mycorrhizal variants and the C variant. The P content was 2 times higher in the mycorrhizal variants than in the C variant. The dry weight of the shoots of *T. medium* in IM was statistically significantly different from that of the other variants. The dry weight of the shoots of *T. medium* in IM and CM was 3 and 2 times higher than in C, respectively.

### 2.2. Mycorrhizal Studies

#### 2.2.1. Microscopic Analysis

In the roots of both plant species, structures characteristic of AMF were observed in the mycorrhizal variants. No mycorrhizal structures were observed in the sterilized controls.

**Figure 1:**
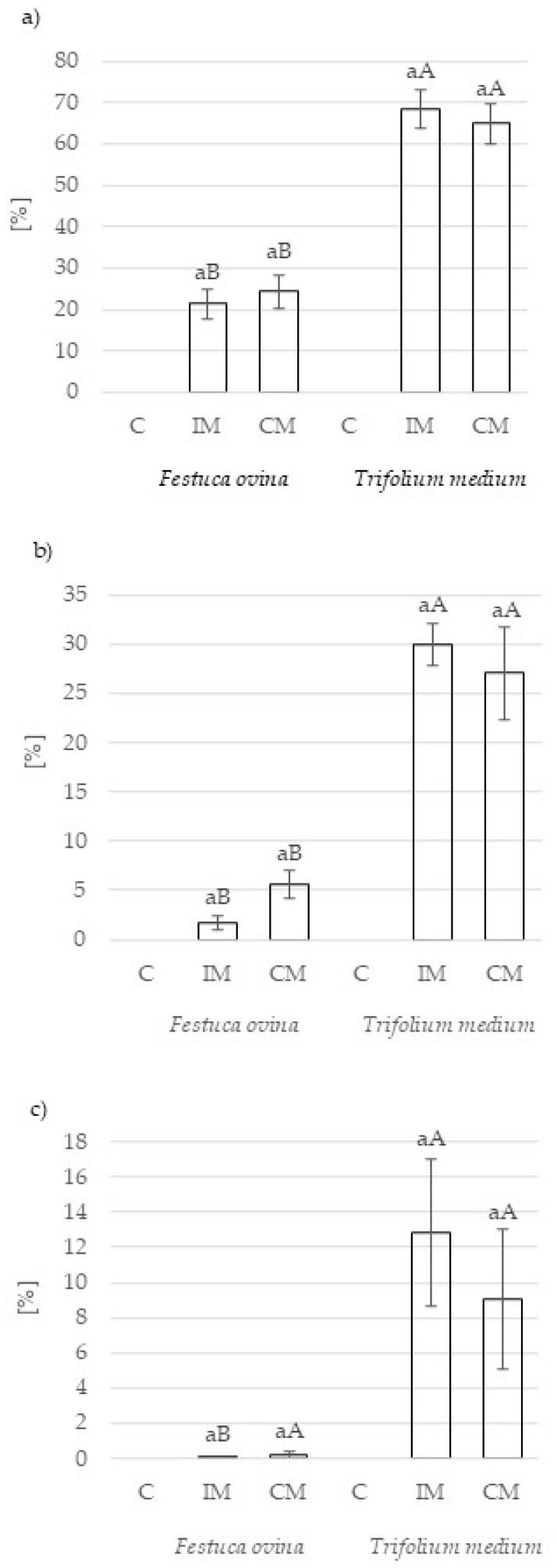
Parameters of mycorrhizal colonization in *Festuca ovina* and *Trifolium medium* roots: (**a**) F—frequency of mycorrhiza in the root system, (**b**) M—relative mycorrhizal intensity in the root system, (**c**) A—relative abundance of arbuscules in the root system, IM—inoculum with mycorrhiza from the calcareous grassland, CM—commercial mycorrhizal inoculum. Values are means ± SE, *n* = 5. Lowercase letters denote significant differences between different experimental treatments and Uppercase letters denote significant differences between species for specific parameters, according to the Fisher LSD test (*p* ≤ 0.05).

No significant differences were found in mycorrhizal frequency in the root system (F), relative mycorrhizal intensity in the root system (M) and relative abundance of arbuscules in the root system (A) when looking at the differences between species in the different inoculation treatments, while no AMF colonization indicators were found in the control treatment. Interestingly, *F. ovina* showed significantly lower values for all measured parameters, and these were about 3 and 6 times higher for F and M, respectively, in *T. medium* than in *F. ovina*. In addition, negligible values were found for the parameter A in *F. ovina* (Figure 1).

#### 2.2.2. The Composition of AMF Communities

**Figure 2:**
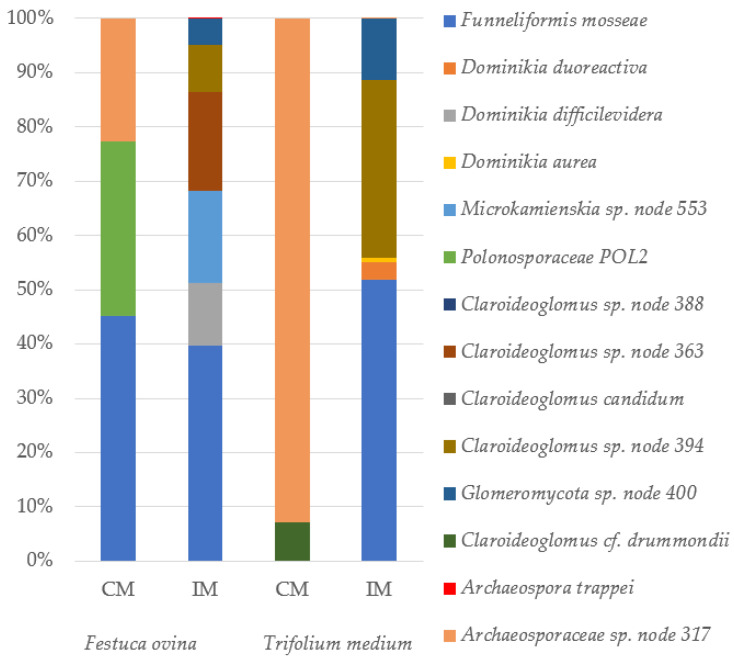
Composition of the AMF communities in *Festuca ovina* and *Trifolium medium* roots. IM—inoculum with mycorrhiza from the calcareous grassland; CM—commercial mycorrhizal inoculum.

Based on the sequencing reads, 2,541,315 contigs were obtained. When filtering for quality and size (<300 bp and >470 bp), a total of 52,628 sequences were removed. A total of 14 AMF species were found in the roots of *F. ovina* and *T. medium* in all samples. The percentage of AMF species identified in the roots of *F. ovina* and *T. medium* with mycorrhizal inoculum from the calcareous grasslands and commercial mycorrhizal inoculum is shown in Figure 2.

In the IM, *Funneliformis* (40% and 52% participation in the roots of *F. ovina* and *T. medium*, respectively) and *Claroideoglomus* (27% and 33% participation in the roots of *F. ovina* and *T. medium*, respectively) were the most abundant genera. In CM, the dominance of *Funneliformis mosseae* (45% participation), *Polonosporaceae* POL2 (32% participation) and *Archaeosporaceae* sp. node 317 (23% participation) was observed in the roots of *F. ovina*. The roots of *T. medium* were dominated by *Archaeosporaceae* sp. node 317 (93% participation). Species such as *Dominikia difficilevidera*, *Microkamienskia* sp. node 553, *Claroideoglomus* sp. node 363 and *Archaeospora trappei* were only detected in the roots of *F. ovina* in IM. In contrast, *Dominikia duoreactiva*, *Dominikia aurea* and *Archaeosporaceae* sp. node 317 were identified only in the roots of *T. medium*. In roots with CM, *F. mosseae* and *Polonosporaceae* POL2 were only present in *F. ovina* (Figure 2).

**Figure 3:**
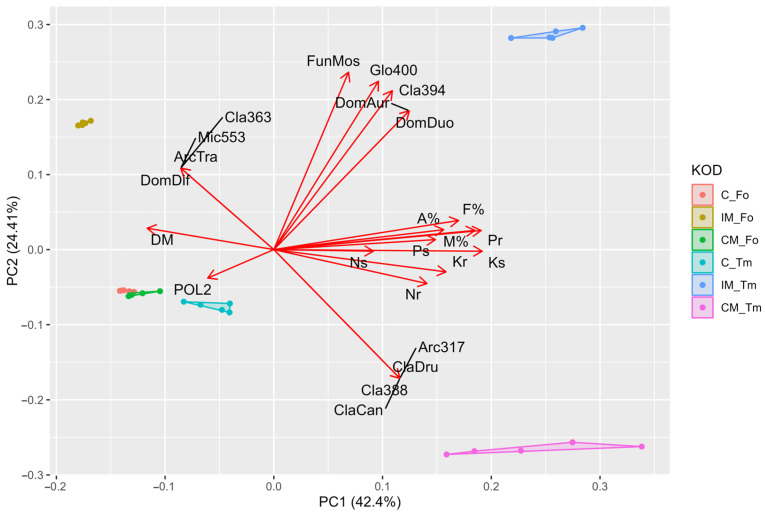
The principal component analysis for *Festuca ovina* (Fo) and *Trifolium medium* (Tm), cultivated in soil substrate without inoculation (C), with inoculum originated from calcareous grassland environment (IM) and with commercially available inoculum (CM). Abbreviations for correlation PCA: DM—shoot dry matter, Ps—P shoot content, Pr—P root content, Ns—N shoot content, Nr—N root content, Ks—K shoot content, Kr—K root content, F%—mycorrhizal frequency, M%—relative mycorrhizal intensity, A%—relative abundance of arbuscules, FunMos—*Funneliformis mosseae* richness, DomDuo—*Dominikia duoreactiva* richness, DomDif—*Dominikia difficilevidera* richness, DomAur—*Dominikia aurea* richness, Mic553—*Microkamienskia* sp. node 553 richness, POL2—*Polonosporaceae* POL2 richness, Cla388—*Claroideoglomus* sp. node 388 richness, Cla363—*Claroideoglomus* sp. node 363 richness, ClaCan—*Claroideoglomus candidum* richness, Cla394—*Claroideoglomus* sp. node 394 richness, Glo400—*Glomeromycota* sp. node 400 richness, ClaDru—*Claroideoglomus* cf. *Drummondii* richness, ArcTra—*Archaeospora trappei* richness and Arc317—*Archaeosporaceae* sp. node 317 richness.

Principal Component Analysis performed for all parameters studied and clustered by treatments confirmed that *F. ovina* has more biomass but performs worse in terms of colonization and nutrient enrichment. Interestingly, the greatest impact of inoculation was found in *T. medium*, where significant improvements in nutrients were found. To support this statement, the gap between the inoculated and non-inoculated *T. medium* treatments was much larger, whereas, in *F. ovina,* the groups were close to each other. The distribution of the community in the PCA could be divided into four groups, each targeting a different corner of the coordinate system. Two groups on the left distinguish the treatment with *T. medium* and two on the right, indicating a higher community richness (upper left corner) in the Fo_IM treatment, and the controls with the CM_Fo treatment show a really low community richness considering the pure number of sequences detected (Figure 3).

## 3. Discussion

The roots of the two tested plant species were colonized by AMF independently of the inoculation treatments; no AM fungal structures were present in the roots of the sterilized control (C). The effect of inoculation was reflected in the values of the AMF colonization parameters (F, M and A). *T. medium* was more colonized compared to *F. ovina* independent of the inoculation variant. Arbuscules were extremely rare, up to 12% in the roots of *T. medium*; in the study by Karanika et al. [42], *T. medium* was also more colonized by AMF than *F. ovina*. Scheublin et al. [43] showed an AMF colonization of about 40% and the presence of arbuscules in the roots of *F. ovina*. The differences between the AMF root colonization levels could be due to the different colonization strategies of the AMF species [44]. Both species studied showed increased biomass under AMF inoculation with commercial (CM) inoculum, but, for inoculum from calcareous grassland (IM), this observation was only true for *T. medium*.

Rusinowski et al. [45] reported that *Zea mays* L. grown in sterilized and non-sterilized soil showed no differences in shoot and root N, P and K content, but shoot biomass was higher in the sterilized treatment. As in this work, the values of colonization parameters were negligible in the sterilized treatments. These differences could be due to two factors. *Z. mays* is a crop that is alien to Polish conditions and has been intensively cultivated for decades [46], moreover, the physicochemical properties of the soil in Rusinowski et al. [45] indicate higher soil P availability, a known factor determining the effect of AMF on plant growth and development [47]. Other reports have also shown that inoculation of AMF favors plant development when P and N are limited [48,49,50], directly reflecting the characteristics of our experimental substrate. Xiao et al. [51] reported that *Trifolium repens* cultivated in sterilized Cd-contaminated soil with and without AMF inoculation did not significantly change the N, P and K content in shoots or shoot and root biomass. This difference from the present study could be mainly related to the presence of Cd, whose negative effect on AMF colonization has been confirmed by other studies [45].

Xu et al. [52] reported that, for *Festuca arundinacea*, the application of different arbuscular endophytes in a pot experiment increased the dry weight of the shoots and the N, P and K content in the shoots and roots of the investigated plant compared to the non-inoculated control. These results confirm the results of the experiment as the application of inoculants increased the measured parameters and one of the inoculants tested was based on *F. mosseae*. A strong dominance of *F. mosseae* was observed in the roots of both the mycorrhizal-treated plants from calcareous grassland and *F. ovina* in the CM treatments. There are also other reports confirming the utilitarian character of *F. mosseae*. Keshavarz et al. [53] reported that this strain had positive effects on the growth of vetiver grass in calcareous soil. Joner et al. [54] also showed increased growth of *Trifolium subterraneum* after inoculation of *F. mosseae*. In addition, Shi et al. [55] showed that *F. mosseae* improved the N, P and K uptake of wheat plants in soils with limited nutrients. Moreover, *F. mosseae* is able to tolerate stress factors such as metal contamination, salinity, drought and low temperature [56,57]. All this confirms the importance of *F. mosseae* as a component of inoculants that can be used for a variety of grassland and crop species.

Shi et al. [55] showed a correlation between the increase in biomass and the increase in N and P content in shoots and roots of plants after inoculation with AM fungi. In the *T. medium*, both inoculation variants improved P and K uptake, confirming the hypothesis that inoculation with AM fungi, which promotes P and K uptake, also promotes plant biomass. Similar results were observed in the study by Sui et al. [58] for *T. repens*. Interestingly, the N content in both roots and shoots was not affected by IM nor CM inoculum. On the one hand, Courty et al. [59] and Koegel et al. [60] reported that improved N uptake was observed after the application of AMF inoculum, while the previously described work by Xiao et al. [51] for *T. repens* suggests that this positive effect is absent. In this study, *F. ovina* inoculated with CM was the only experimental treatment in which a significant increase in N uptake in root and shoot was confirmed. It seems that in *F. ovina* the N content is the main factor contributing to the increase in shoot dry weight.

Mycorrhiza may play a key role in the performance of plants in nature-based solutions such as green roofs with designed soils under harsh environmental conditions. Recent studies have shown that even different AMF isolates belonging to the same AMF species can induce very different responses to plant growth [43,61]. Contrasting results regarding plant growth on green roofs after inoculation with AM fungi were found by Schroeder et al. [62], where inoculation with commercial mycorrhizae (53% *Rhizoglomus irregulare*, 27% *F. mosseae* and 20% *Funneliformis caledonium*) increased the growth of 9 of the 11 plants tested by 2.5-fold.

Despite the results for biomass and nutrient content of *T. medium* and *F. ovina*, both species show higher AMF species richness under IM inoculation. Turrini et al. [63] reported that a higher number of OTUs was found in treatments with high-diversity cover crops on agricultural land than in treatments with low-diversity cover crops. These results reflect the difference mentioned above, in that, the more diverse the source material for inoculation, the greater the diversity and richness in the AMF root community. Moreover, in this work, *F. ovina* showed higher community richness compared to the same *T. medium* inoculation treatments. This observation was also confirmed by Scheublin et al. [64] after collecting the plants grown on different turf samples from the environment. Kobae [65] pointed out that the benefits of different AMF species may vary, which could be due to differences in abundance, interactions with plants and competition between fungi [38]. This observation is reflected in the current studies, as each species favors a different inoculation, especially at shoot dry weight.

All of the above suggests that the response of plants to AMF inoculation depends on many factors including, in these cases, soil fertility and species composition of AMF communities in the root system. For this reason, more field trials and more inoculants need to be investigated with regard to the application of different inoculants for calcareous grass species grown in green city infrastructure.

## 4. Materials and Methods

### 4.1. Experimental Design

The soil substrate was designed to reflect the conditions on roofs and walls, i.e., low nutrient content and the requirements for sufficient infiltration capacity. The soil substrate for the pot experiment consisted of 65% technical sand (granulation 0–1.8 mm) (*w*/*w*), 10% garden compost (*w*/*w*) and 25% soil (sandy clay) (*w*/*w*) collected from the calcareous grassland near Bytom, Poland. The soil substrate was mixed manually [15].

Three samples from compost and substrate were collected, air-dried and sieved for future analysis. Soil samples were analyzed for texture, pH and electrical conductivity (EC) using standardized methods. The analysis of the soil texture was carried out using the areometric method according to the PN-R-04032 standard [66]. Soil pH was measured in H_2_O and 1 M KCl (ratio 1:2.5 *m*/*v*) using a combined glass/calomel electrode (OSH 10–10, METRON, Gliwice, Poland) and a pH meter (CPC-551, Elmetron, Gliwice, Poland) at 20 °C. Electrical conductivity was determined with an ESP 2ZM electrode (EUROSENSOR, Gliwice, Poland) according to the Polish standard [67]. Total nitrogen concentration in soil was measured using the Kjeldahl method [68] according to the standard PN-ISO 11261:2002 [69]. Potassium was measured by flame atomic absorption spectrometry (iCE 3500 FAAS, Thermo Scientific, Cambridge, UK) and phosphorus by ICP (Liberty 220, Varian, Palo Alto, USA). The content of available potassium (K_2_O) and phosphorus (P_2_O_5_) were measured according to Egnér et al. (1960) [70]. Organic carbon was determined according to the Tiurin method (Nelson and Sommers 1996) [71]. Soil organic matter (SOM) was measured using the loss on ignition method as follows: the soil was dried at 105 °C for 24 h and then treated (5 g) at 550 °C for 4 h [72].

The physicochemical properties of the compost and soil substrate are listed in Table 2.

To remove the autochthonous AMF contained in the soil, the substrate was sterilized three times in an autoclave. The sterilization parameters were as follows: a temperature of 121 °C, pressure of 205 kPa and time of 30 min [73]. Pots with a volume of 3 L were filled with 2.75 kg of the substrate. The experimental variants were as follows: C—without mycorrhiza, IM—inoculum with mycorrhiza from the calcareous grassland and CM—commercial mycorrhizal inoculum.

Two variants of mycorrhizal inoculum were used for the experiment: commercial mycorrhiza AM Symbivit (Symbiom Ltd., Lanskroun, Czech Republic) and mycorrhiza collected from calcareous grassland in the Silesia region (Poland). The mycorrhizal inoculum was prepared by collecting grass roots from a depth of 0–20 cm from calcareous grassland in Katowice, where native plants were indentified. The mycorrhizal inoculum was prepared according to [74] using *Lolium perenne* as a host plant. Sequencing of indigenous inoculum amplified using the FULFN1ngs/FULFN2ngs–FULRNngs primers including the adaptors for sequencing on the MiSeq platform (Illumina, San Diego, CA, USA) revealed the following AMF species: *F. mosseae* (58%), *Septoglomus* sp. node 508 (42%) and other species (<1%): *Funneliformis* sp. node 446, *Septoglomus* sp. node 514, *D. duoreactiva*, *D. difficilevidera*, *D. aurea*, *Microkamienskia* sp. node 553, *Claroideoglomus* sp. node 363, *Claroideoglomus* sp. node 394, *Glomeromycota* sp. node 400 and *A. trappei* (unpublished data). A total of 42 g of the inoculum was mixed with the top layer (3 cm—400 g soil) of the soil substrate in each pot [75]. A total of four plants per pot were grown from surface-sterilized seed [76] of either commercial *F. ovina* and wild-harvested seeds of *T. medium* with a total of 30 pots (2 species × 3 treatments × 5 replicates). Plants were grown for 6 months in a phytotron under controlled conditions: temperature 22/16 °C (day/night), light intensity PAR = 300 μmol (photons) m^−2^ s^−1^, photoperiod 16/8 h and relative humidity around 40% [15]. At the end of the experiment, samples of the shoots and roots were taken for further analysis.

### 4.2. Plant Sampling and Preparation for Future Analysis

The shoots and roots for the estimation of plant biomass and NPK content were washed with tap water and dried at 70 °C for 3 days. After a constant weight was reached, the dry weight was determined. The samples were ground to a homogeneous powder (<1 mm) for further analysis. The roots for microscopic observation were stored in 50% ethyl alcohol at 4 °C. To determine the species composition of the AMF, the roots were stored at −20 °C until DNA extraction.

### 4.3. NPK Content in Plant Tissues

To determine the total nitrogen (N) concentration, the shoot and root samples were digested with H_2_SO_4_. After the addition to the digested samples of 10 M NaOH, the samples were distilled with H_3_BO_3_. Then, the total nitrogen (N) concentration in the samples was determined using the titration method using standard 0.005 M H_2_SO_4_ [77]. To determine the total phosphorus (P) and potassium (K) concentration, the samples were first mineralized with concentrated HNO_3_ and H_2_O_2_ (4:1 *v*/*v*) and then measured with ICP (Liberty 220, Varian, Palo Alto, CA, USA).

### 4.4. Estimation of AMF Colonization in Roots

#### 4.4.1. Microscopic Analysis of AMF in Roots

The roots were prepared for microscopic observations according to the method of Phillips and Hayman [78]. The roots were first cleaned in 7% KOH for 24 h, rinsed in deionized water and acidified in 5% lactic acid for the next 24 h. They were then stained with 0.05% aniline blue in lactic acid for 24 h. Microscopic observations were performed under a Zeiss Axio Imager D2 fluorescence microscope (Zen 2 software, Zeiss, Germany). AMF colonization was assessed according to Trouvelot [79]. The parameters were used to describe the characteristics of mycorrhizal structure development: mycorrhizal frequency in the root system (F%), relative mycorrhizal intensity in the root system (M%) and relative abundance of arbuscules in the root system (A%) were calculated using MYCOCALC software [80] according to formulas as follows:

$$F\%=\frac{number\;of\;roots\;fragments\;with\;mycorrhiza}{N}*100$$

$$M\%=\frac{95n5+70n4+30n3+5n2+n1}{N}$$

$$m\%=\frac{M\%*N}{number\;of\;roots\;fragments\;with\;mycorrhiza}$$

$$a\%=\frac{100mA3+50mA2+10mA1}{100}$$

$$A\%=\frac{a\%}{\left(M\%/100\right)}$$

Abbreviations: *N*—total number of roots fragments; n5, n4, n3, n2, n1—number of roots fragments rated as 5, 4, 3, 2, 1; m%—intensity of the mycorrhizal colonization in the root fragments, a%—arbuscule abundance in mycorrhizal parts of root fragments, mA3, mA2, mA1—% fragments of roots with mycorrhiza rates as A3, A2, A1, for example, $A3=\frac{95n5A3+70n4A3+30n3A3+5n2A3+n1A3}{number\;of\;roots\;fragments}*\frac{100}{m\%}$.

#### 4.4.2. Molecular Analysis of AMF Communities in Roots

The DNA from the roots was isolated using the Wizard^®^ Genomic DNA Purification Kit (Promega, Madison, WI, USA) and the concentration in the samples was measured using a Eppendorf BioSpectrometer^®^ fluorescence (Eppendorf AG, Hamburg, Germany). For PCR, we used the following reagents: Color Taq PCR Master Mix (EURx^®^, Gdańsk, Poland), 8 μL of 5 mM MgCl_2_, 4 μL of BSA and set of primers SSUmAf/LSUmAr [81], in a total volume of 20 µL. The PCR reaction was performed in a Mastercycler^®^ nexus GSX1 (Eppendorf AG, Hamburg, Germany) under the following thermal cycling conditions: 95 °C for 5 min; 34 cycles: denaturation at 95 °C for 20 s, 51 °C for 30 s annealing, 72 °C for 1 min 45 s elongation; 72 °C for 10 min final elongation. The quality of the PCR products was resolved by electrophoresis in a 1.5% agarose gel (Basica LE GQT Prona Agarose, Resolva GQT Prona Agarose; 1:1; *w*/*w*). The PCR products were used as a template for nested PCR. The nested PCR was performed with the Color Taq PCR Master Mix (EURx^®^, Gdańsk, Poland) and the primers FULFN1ngs/FULFN2ngs–FULRNngs including the adaptors for sequencing on the MiSeq platform (Illumina, San Diego, CA, USA) in a total volume of 50 µL. PCR conditions were as follows: 95 °C for 1 min, 35 cycles of: 95 °C for 20 s, 58 °C for 25 s, 72 °C for 45 s, 72 °C for 2 min and final elongation at 12 °C [82]. The quality of the PCR products was resolved by electrophoresis as before and yielding 1 band per sample below 500 bp (the pool of *Glomeromycota* amplicons) (Figure S1). The Clean-up Kit (A&A Biotechnology, Gdynia, Poland) was used to purify the PCR products. Paired-end (PE) technology, 2 × 300 nt, with the Illumina v3 kit and library indexing with the Nextera kit was ordered from Genomed S.A. (Warsaw, Poland).

#### 4.4.3. Bioinformatic and Statistical Analysis

The obtained sequences were trimmed with Cutadapt 3.0. By using the Mothur 1.48, the sequences were merged into contigs. Subsequently, sequences with less than 300 bp, more than 470 bp, more than four ambiguous sequences, homopolymers with more than 22 nucleotides and potentially chimeric sequences were removed [83]. To group the sequences into clusters, the DGC (Distance-based greedy clustering) method with a cutoff = 0.02 was used. The sequences were aligned to the reference sequence dataset using the MAFFT program available on XSDE 7.305 [84] and the following parameters: Global ordering of sequences was performed using the auto-strategy, scoring matrix for nucleotide sequences 200 PAM/k = 2. Taxonomic identification was performed using the RAxML-EPA tool with GTRGAMMAI nucleotide substitution model [85]. The output was used for taxonomic annotation of the sequences in Gappa v0.8.0 [86] and analyzed with the Archaeopteryx Tree Viewer. In addition, the sequences were compared with sequence databases (NCBI, National Center for Biotechnology Information) using BLAST [82,87].

Content of NPK, plant biomass and mycorrhizal colonization parameters were analyzed using Statistica 13.1 (Dell, Round Rock, TX, USA). The normal distribution within the data set was determined using the Kolmogorov–Smironov test. The significance of differences was tested by one-way ANOVA with LSD post hoc test (test experimental treatments, *p* < 0.05).

## 5. Conclusions

The conclusion is contrary to the hypothesis that the biomass yield of *F. ovina* was higher in the CM treatment, while *T. medium* reached higher values in the IM treatment, indicating a species-specific response to inoculation in terms of shoot dry weight. The hypothesis on NPK content was negatively confirmedbecause treated by CM resulted in higher nutrient levels. The last hypothesis was confirmed, as more species were found in the IM-inoculated plants regardless of the species studied. Nevertheless, N content was the most important parameter causing the increase in biomass of *F. ovina. T. medium* showed higher colonization rates compared to *F. ovina* regardless of the treatment and *F. ovina* showed higher community richness in the respective treatments compared to *T. medium*.

## Figures and Tables

**Table 1:** Biomass and NPK content in shoots and roots of *Festuca ovina* and *Trifolium medium*.

Variant	C	IM	CM
Shoots	*Festuca ovina*		
P [mg kg^−1^]	1019 ± 23 b	1086 ± 23 ab	1096 ± 59 a
K [mg kg^−1^]	7800 ± 196 b	8697 ± 252 b	12,366 ± 420 a
N [%]	0.64 ± 0.01 b	0.63 ± 0.01 b	1.01 ± 0.02 a
Dry weight [g]	5.64 ± 0.20 b	6.13 ± 0.14 b	9.55 ± 0.41 a
Roots			
P [mg kg^−1^]	980 ± 12.4 a	973 ± 8.6 a	1029 ± 36 a
K [mg kg^−1^]	2293 ± 102 b	4342 ± 490 a	3472 ± 138 a
N [%]	0.86 ± 0.0 ab	0.76 ± 0.1 b	0.94 ± 0.0 a
Shoots	*Trifolium medium*		
P [mg kg^−1^]	483.98 ± 13.1 b	2145 ± 324 a	2344 ± 357 a
K [mg kg^−1^]	12,008 ± 530 b	28,990 ± 1224 a	29,779 ± 1409 a
N [%]	2.12 ± 0.54 a	2.03 ± 0.14 a	2.33 ± 0.16 a
Dry weight [g]	1.10 ± 0.04 b	3.01 ± 0.40 a	2.17 ± 0.49 ab
Roots			
P [mg kg^−1^]	1135 ± 42 b	2200 ± 95 a	2049 ± 155 a
K [mg kg^−1^]	10,721 ± 459 b	10,810 ± 601 b	12,067 ± 511 a
N [%]	1.70 ± 0.2 a	1.75 ± 0.2 a	1.87 ± 0.3 a

The values are mean ± SE, *n* = 5. Lower case letters (a, b) indicate significant differences between the experimental variants when differences within a plant species are taken into account, according to Fisher LSD test (*p* ≤ 0.05). C—control; IM—inoculum with mycorrhiza from calcareous grassland; CM—commercial mycorrhizal inoculum.

**Table 2:** Physicochemical parameters and concentration of elements in soil substrate.

		Value	
Parameter		Compost	Substrate
Texture		-	Loamy sand
pH	H_2_O	7.43 ± 0.02	8.25 ± 0.03
	KCl	6.99 ± 0.00	7.99 ± 0.01
EC [µS cm^−1^]		531.7 ± 21.85	190.12 ± 8.15
Corg [%]		7.14 ± 1.41	1.79 ± 0.19
SOM [%]		29.67 ± 0.06	3.97 ± 0.19
P [mg kg^−1^ d.w.]		1499 ± 76.85	498.373 ± 24.07
K [mg kg^−1^ d.w.]		3920 ± 190	1117 ± 53.72
N [mg kg^−1^ d.w.]		0.76 ± 0.21	0.190 ± 0.0
P_2_O_5_ [mg 100 g^−1^ d.w.]		22.287 ± 1.35	0.476 ± 0.14
K_2_O [mg 100 g^−1^ d.w.]		343.267 ± 18.89	42.996 ± 5.51

Values are mean ± SE, *n* = 3. Abbreviations: EC—Electrical Conduction, SOM—soil organic matter, P_2_O_5_—available phosphorus, K_2_O—available potassium.

## Data Availability

The original contributions presented in this study are included in the article and Supplementary Material; further inquiries can be directed to the corresponding author.

## Supplementary Materials

The following supporting information can be downloaded at: https://www.mdpi.com/article/10.3390/plants13182620/s1. Figure S1: Fragment of an agarose gel with purified DNA using the Perfect™ 100–1000 bp DNA ladder (EURx^®^, Gdańsk, Poland).
