# Supplementary material for: Inoculation with Arbuscular Mycorrhizal Fungi Supports the Uptake of Macronutrients and Promotes the Growth of Festuca ovina L. and Trifolium medium L., a Candidate Species for Green Urban Infrastructure
Source: Plants (Basel). 2024 Sep 19;13(18):2620. doi: 10.3390/plants13182620 (PMC11434852; doi:10.3390/plants13182620)
Supplement: Supplementary file 1 [file plants-13-02620-s001.zip › plants-3160475-supplementary.pdf]

Supplementary materials:

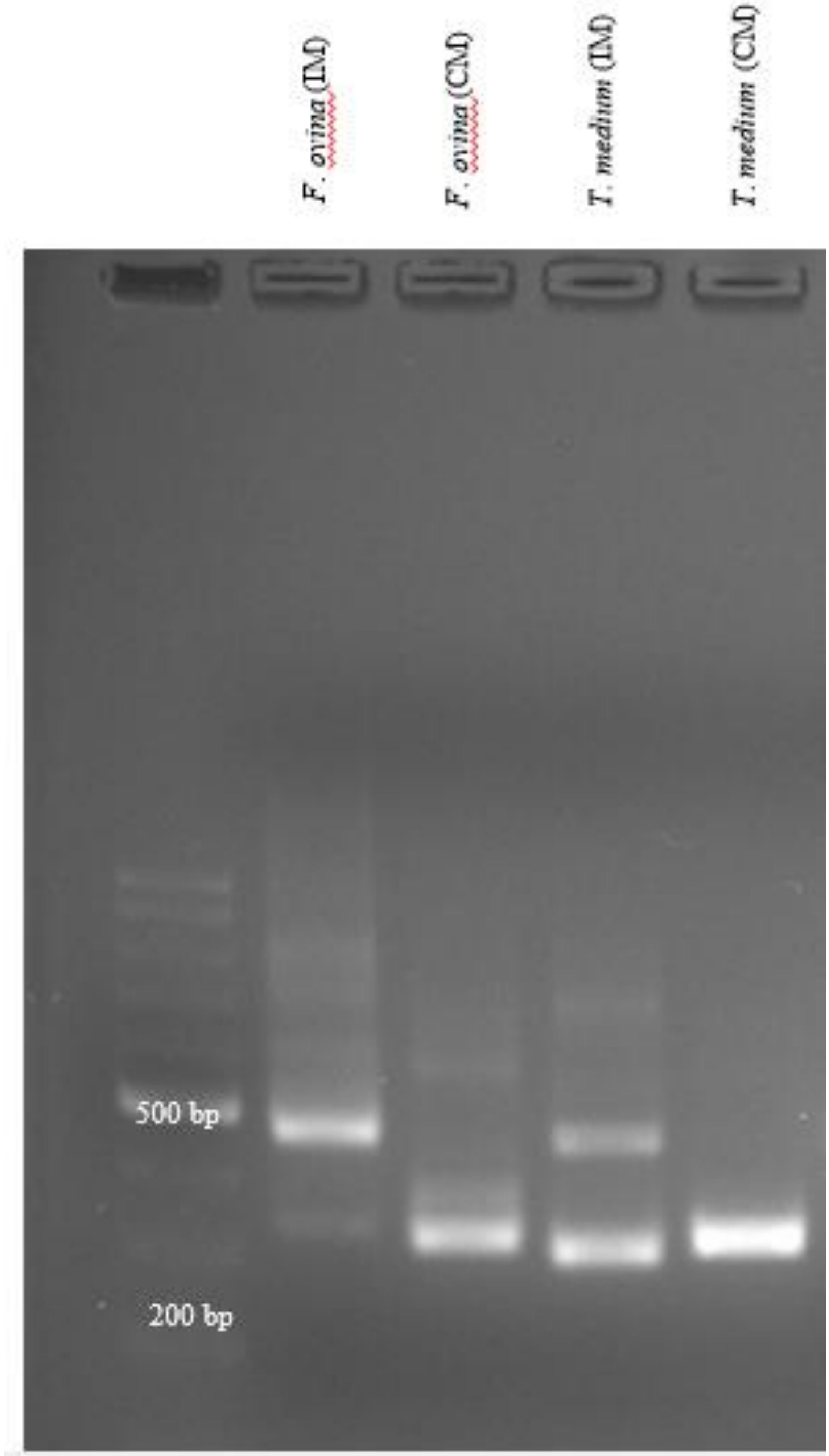

Figure S1. Fragment of an agarose gel with purified DNA using the Perfect™ 100-1000 bp DNA ladder (EURx®). <500 bp there are products containing LSU rDNA sequences belonging to Glomeromycotina. *F. ovina* and *T. medium*. IM – inoculum with mycorrhiza from calcareous grassland, CM – commercial mycorrhizal inoculum.
